# Synthesis of Some New Pyridazine Derivatives for Anti-HAV Evaluation

**DOI:** 10.3390/molecules22010148

**Published:** 2017-01-17

**Authors:** Eman M. Flefel, Waled A. Tantawy, Walaa I. El-Sofany, Mahmoud El-Shahat, Ahmed A. El-Sayed, Dina N. Abd-Elshafy

**Affiliations:** 1 Department of Chemistry, College of Science, Taibah University, Al-Madinah Al-Monawarah 1343, Saudi Arabia; 2 Department of Photochemistry, Chemical Industries Research Division, National Research Centre, 33 EL-Bohouth St., Dokki 12622, Giza, Egypt; walednrc@gmail.com (W.A.T.); walaa.elsofany@gmail.com (W.I.E.-S.); mahmoudelshahat@gmail.com (M.E.-S.); ahmedcheme4@yahoo.com (A.A.E.-S.); 3 Department of Water Pollution, Environmental Research Division, National Research Centre, 33 EL-Bohouth St., Dokki 12622, Giza, Egypt; dnanadeem@yahoo.com

**Keywords:** pyridazine, fused pyridazine derivatives, hepatitis A virus (HAV)

## Abstract

4-(2-(4-Halophenyl)hydrazinyl)-6-phenylpyridazin-3(2*H*)-ones **1a**,**b** were prepared and treated with phosphorus oxychloride, phosphorus pentasulphide and ethyl chloroformate to give the corresponding chloropyridazine, pyridazinethione, oxazolopyridazine derivatives **2**–**4**, respectively. Compound **2** reacted with hydrazine hydrate to afford hydrazinylpyridazine **7**. The reaction of 4-(2-(4-chlorophenyl)hydrazinyl)-3-hydrazinyl-6-phenylpyridazine (**7**) with acetic anhydride, *p*-chlorobenzaldehyde and carbon disulphide gave the corresponding pyridazinotriazine derivatives **8**–**10**. On the other hand, 5-(4-chlorophenylamino)-7-(3,5-dimethoxybenzylidene)-3-phenyl-5*H*-pyridazino[3,4-*b*][1,4]thiazin-6(7*H*)-one (**11**) was prepared directly from the reaction of compound **3** with chloroacetic acid in presence of *p*-chlorobenzaldehyde. Compound **11** reacted with nitrogen nucleophiles (hydroxylamine hydrochloride, hydrazine hydrate) and active methylene group-containing reagents (malononitrile, ethyl cyanoacetate) to afford the corresponding fused compounds **12**–**15**, respectively. Pharmacological screening for antiviral activity against hepatitis A virus (HAV) was performed for the new compounds. 4-(4-Chlorophenylamino)-6-phenyl-1,2-dihydropyridazino[4,3-*e*][1,2,4]triazine-3(4*H*)-thione (**10**) showed the highest effect against HAV.

## 1. Introduction

**Figure 1:**
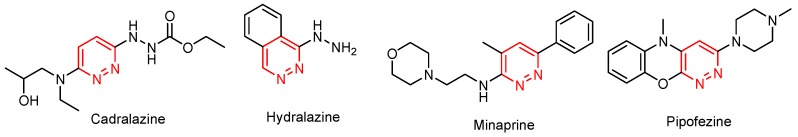
Some example of common drugs based on pyridazine rings.

Heterocyclic compounds based on the pyridazine backbone have been cited as significant biologically active pharmacophores in the field of medicinal chemistry, providing a wide range of safe and effective drugs [1,2,3,4,5,6]. The pyridazine ring is thus a part of the structures of some therapeutic agents available on the market like cadralazine [7,8], minaprine [9,10,11], hydralazine [12,13], pipofezine [14], etc. (Figure 1).

Pyridazine-based frameworks are widely distributed in biologically active products with anti-viral [15,16,17,18,19,20], anti-inflammatory [21,22,23,24,25], anti-microbial [26,27,28], anti-cancer [29,30,31,32,33,34,35,36], anti-convulsant [37,38,39,40], anti-analgesic [41,42], anti-tubercular [43,44,45], anti-hypertensive [3,46], anti-diabetic [47], anti-depressant [48,49], anti-Alzheimer’s [50,51], phosphodiesterase [52,53], platelet aggregation [54,55,56,57], and cholesterol acyl transferase inhibitor properties [58]. Peptide nucleic acids (PNAs) with new pyridazine-type nucleobases were reported as replacements for the DNA duplex bases instead of thymine, adenine, guanine and cytosine to form novel DNA or RNA duplexes [59]. Hepatitis A is a highly contagious liver infection caused by the hepatitis A virus (HAV); it is usually transmitted by the fecal-oral route, either through person-to-person contact or consumption of contaminated food or water [60]. Hepatitis A is a self-limited disease that does not result in chronic infection. Tens of millions of infections are reported each year, with a higher incidence in developing countries. HAV infection causes fever, malaise, weakness, anorexia, nausea, vomiting, arthralgias and myalgias [61,62]. Although there are no commercial antiviral drugs specifically licensed for treating HAV infections, ribavirin, amentadine and 2-deoxy-d-glucose are among several antiviral substances known to interfere with HAV replication [63]. Many heterocyclic compounds were reported as anti HAV agents [64,65,66,67]. In view of these observations much attention has been devoted to develop novel pyridazines-based drug candidates. We report herein the synthesis of some new heterocyclic compounds containing pyridazine moieties and the study of their anti-viral activities compared to amentadine as positive control.

## 2. Results and Discussion

### 2.1. Chemistry

**Scheme 1:**
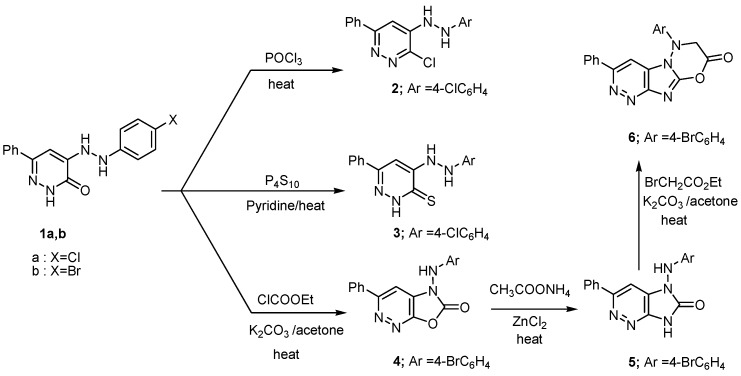
General methods for preparation of compounds **2**–**6**.

The 4-(2-(4-halophenyl)hydrazinyl)-6-phenylpyridazin-3(2*H*)-one compounds **1a,b** were prepared by refluxing 3-[2-(4-halophenyl)hydrazono]-5-phenylfuran-2(3*H*)-ones with hydrazine hydrate in absolute ethanol [68]. Compound **1a** was reacted with phosphorus oxychloride to afford the 3-chloropyridazine derivative **2**. The IR spectrum of compound **2** revealed the absence of a C=O signal. The reaction of compound **1a** with phosphorus pentasulphide afforded pyridazinethione derivative **3**. The same pyridazinethione **3** was also obtained when the 3-chloropyridazine derivative **2** was reacted with thiourea via the reaction of an unisolated thiouronium salt. The IR spectrum of pyridazinethione **3** showed the absence of a C=O group and the presence of a characteristic C=S group band at 1228 cm^−1^. On the other hand, when compound **1b** was treated with ethyl chloroformate in the presence of anhydrous potassium carbonate it afforded the oxazolopyridazine **4**. This was treated with ammonium acetate in the presence of ZnCl_2_ to give imidazopyridazinone **5**. Heating a mixture of imidazopyridazinone **5** with ethyl bromoacetate in the presence of anhydrous potassium carbonate in acetone produced the pyridazinoimidazo oxadiazinone **6** (Scheme 1). The ^1^H-NMR spectrum of compound **6** showed signals at 4.09 (d, *J* = 11.63 Hz, 1H, CH_2_), 4.19 (d, *J* = 11.71 Hz, 1H, CH_2_) and the mass spectrum showed the expected molecular ion peak at *m/z* (%) 421 (11).

When 3-chloropyridazine derivative **2** was refluxed with hydrazine hydrate it afforded the hydrazinopyridazine derivative **7**. The IR and ^1^H-NMR spectra of the latter compound revealed the presence of an NH_2_ group. The potential importance of compound **7** as an interesting intermediate induced us to explore its utility in the synthesis of new fused heterocyclic compounds.

**Scheme 2:**
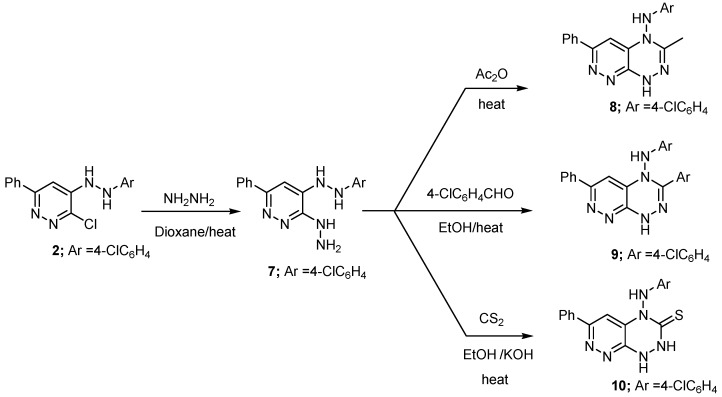
General methods for preparation of compounds **7**–**10**.

Thus, its reactions with acetic anhydride, *p*-chlorobenzaldehyde and carbon disulphide afforded the corresponding pyridazinotriazine derivatives **8**–**10**, respectively (Scheme 2). The spectra of compounds **8**–**10** revealed the absence of the corresponding NH_2_ group bands present in the parent hydrazinopyridazine derivative **7**.

**Scheme 3:**
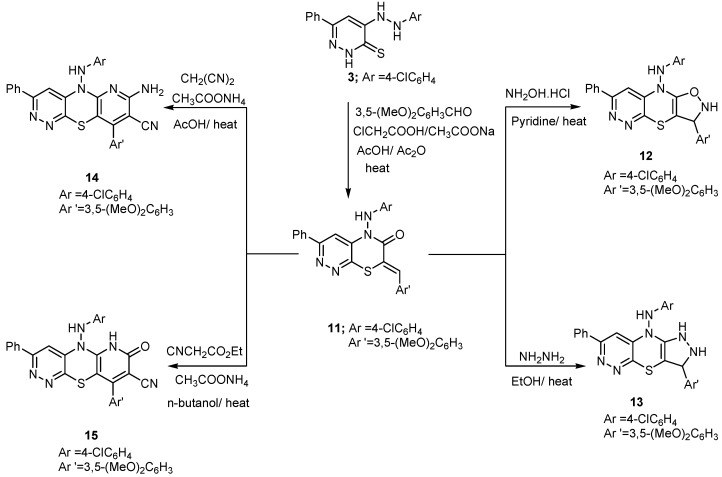
General methods for preparation of compounds **11**–**15**.

Moreover, 5-(4-chlorophenylamino)-7-(3,5-dimethoxybenzylidene)-3-phenyl-5*H*-pyridazino[3,4-*b*][1,4]thiazin-6(7*H*)-one (**11**) was prepared directly from pyridazinethione derivative **3** in a one-pot reaction by reaction with chloroacetic acid, *p*-chlorobenzaldehyde and anhydrous sodium acetate in a mixture of acetic acid/acetic anhydride. The IR spectrum of compound **11** showed the absence of a C=S band and presence of a characteristic C=O group band at 1697 cm^−1^, while the ^1^H-NMR spectrum showed a signal at δ 8.33 (s, 1H, CH, exocyclic-H) (cf. Materials and Methods Section). Finally, compound **11** was used as a key starting material to synthesize a variety of fused heterocyclic compounds. The reaction of compound **11** with nitrogen nucleophiles, namely hydroxylamine hydrochloride and hydrazine hydrate and active methylene group reagents, namely malononitrile, and ethyl cyanoacetate, afforded the corresponding fused compounds **12**–**15**, respectively (Scheme 3). The structure of these compounds was determined on the basis of elemental analysis and spectral data. The IR spectra of compounds **12** and **13** showed the absence of C=O bands and the ^1^H-NMR of compound **14** revealed signals for two OCH_3_ groups at δ 3.80 and 3.81 and an NH_2_ group at 5.3 (s, 2H, NH_2_, D_2_O exchangeable). The IR spectrum showed absorption bands for NH_2_ (3355 cm^−1^), and CN (2222 cm^−1^) groups. On the other hand, The IR spectrum of compound **15** showed absorption bands for C=O (1680 cm^−1^), CN (2221 cm^−1^) and 2NH (3130 and 3166) groups (cf. the Materials and Methods Section).

### 2.2. Antiviral Screening

In the present work we also report the anti-HAV activity results of the novel synthesized compounds. First, a cytotoxicity test was performed to determine safe doses that could be used in the antiviral assays without harming the HEPG2 cells (Table 1).

**Figure 2:**
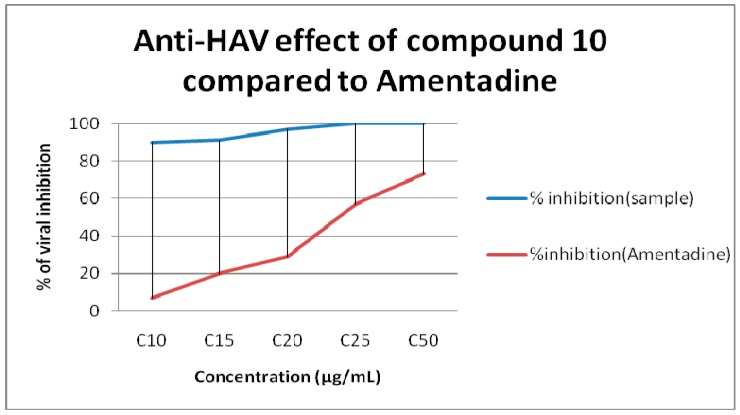
Anti HAV activity of compound **10** compared to amentadine as positive control, C: concentration.

Safe concentrations were used in a plaque reduction assay which was made to detect any changes in viral count as a result of being treated with the compounds with respect to untreated control. Results (Table 2) showed that compound **10** has high antiviral activity against HAV on comparing the effect of different concentrations of compound **10** with the same concentrations of amentadine (positive control). The results (Figure 2) showed that compound **10** has higher inhibitory effect than amentadine at the five concentrations used.

**Figure 3:**
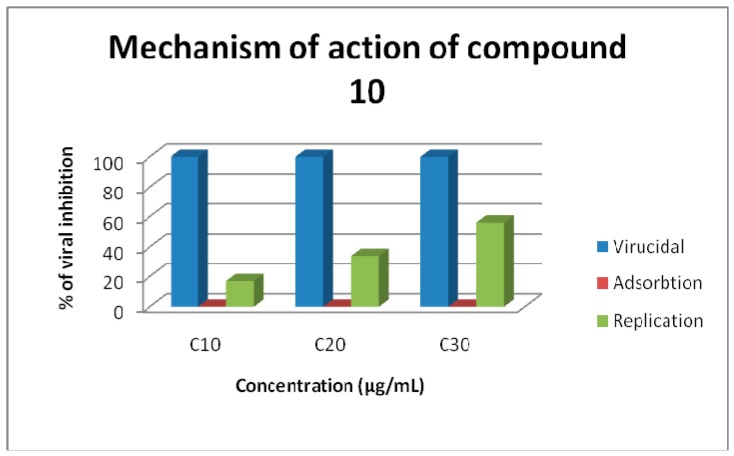
Virucidal effect on viral replication.

The possible mechanism of action of compound **10** was also studied and the results (Figure 3) showed that it has a high virucidal effect and it also shows a mild effect on viral replication. This can be explained by the notion that compound **10** was able to bind to the HAV capsid and thus change its configuration, causing an inability of the virus to bind to its cell receptor, or the compound might also react with the proteins of the capsid also resulting in its inactivation. For its mild effect on replication, this might be due to a mild inhibitory effect of the compound on one or more enzymes needed by the virus to complete its replication cycle, and as a result, showing the observed decrease in viral count. On the other hand the compound has no effect on viral adsorption.

## 3. Materials and Methods

### 3.1. General Inforation

Melting points were measured using an Electrothermal 9100 digital melting point apparatus (Büchi, Flawil, Switzerland) and are uncorrected. IR spectra were recorded on a Perkin-Elmer 1600 FTIR (Perkin-Elmer, Waltham, MA, USA) in KBr discs. ^1^H-NMR spectra (300 MHz) were measured on a Jeol 270 MHz spectrometer (Jeol, Tokyo, Japan) or an Avance spectrometer (Bruker, Karlsruhe, Germany) in DMSO-*d*_6_, and chemical shifts were recorded in δ ppm relative to the internal standard TMS. The mass spectra were run at 70 eV with a Finnigan SSQ 7000 spectrometer (Thermo Electron Corporation, Madison, WI, USA) using EI and the values of *m/z* are indicated in Dalton. Elemental analyses were performed on a Perkin-Elmer 2400 analyzer (Perkin-Elmer) and were found within ±0.4% of the theoretical values. Reaction monitoring and verification of the purity of the compounds was done by TLC on silica gel-precoated aluminum sheets (type 60 F254, Merck, Darmstadt, Germany). All solvents and chemical reagents were purchased from Aldrich (Munich, Germany).

### 3.2. Chemistry

#### 3.2.1. 3-Chloro-4-(2-(4-chlorophenyl)hydrazinyl)-6-phenylpyridazine (**2**)

A mixture of **1a** (0.01 mol) and phosphorus oxychloride (10 mL) was refluxed for 4 h. The mixture was cooled, poured onto crushed ice and then neutralized with 4% NaOH. The precipitate was collected by filtration, washed with water and recrystallized from benzene to give compound **2**. Yield 86%, m.p. 179–181 °C; IR (ν, cm^−1^): 3140, 3153 (2NH); ^1^H-NMR (δ ppm): 4.35 (s, 1H, NH; D_2_O exchangeable), 4.40 (s, 1H, NH; D_2_O exchangeable), 6.64 (s, 1H, pyridazine-H), 6.71–7.97 (m, 9H, Ar-H); MS, *m/z* (%): 330 (M^+^, 68), 332 (M^+^ + 2, 41), 334 (M^+^ + 4, 7). Analysis for C_16_H_12_Cl_2_N_4_ (331.21): calcd. C, 58.02; H, 3.65; Cl, 21.41; N, 16.92. Found: C, 57.83; H, 3.39; Cl, 21.08; N, 16.59.

#### 3.2.2. 4-(2-(4-Chlorophenyl)hydrazinyl)-6-phenylpyridazine-3(2*H*)-thione (**3**)

*Method A.* A mixture of **1a** (0.01 mol) and phosphorus pentasulphide (0.01 mol) in dry pyridine (20 mL) was refluxed for 5 h. After cooling, the reaction mixture was poured onto ice and neutralized with ammonia solution 30%. The separated solid was filtered off, dried and recrystallized from methanol to give compound **3** with yield 50%.

*Method B.* A mixture of **2** (0.01 mol) and thiourea (0.01 mol) in *n*-propanol (40 mL) was refluxed for 4 h. The formed precipitate was collected and dissolved in 10%NaOH (20 mL). The mixture was filtered off and the filtrate was precipitated with HCl (0.1 N). The solid produced was collected and recrystallized from methanol to give compound identical in all aspects with compound **3** (m.p., mixed m.p., TLC). Yield 30%, m.p. 210–212 °C; IR (ν, cm^−1^): 3190–3140 (3NH), 1228 (C=S);^1^H-NMR (δ ppm): 3.89 (s, 1H, NH; D_2_O exchangeable), 4.4 (s, 1H, NH; D_2_O exchangeable), 6.62 (s, 1H, pyridazine-H), 6.7–7.98 (m, 9H, Ar-H), 8.71(s, 1H, NH; D_2_O exchangeable); MS, *m/z* (%): 328 (M^+^, 62), 330 (M^+^ + 2, 20). Analysis for C_16_H_13_ClN_4_S (328.83): calcd. C, 58.44; H, 3.98; Cl, 10.78; N, 17.04; S, 9.75. Found C, 58.17; H, 3.42; Cl, 10.47; N, 16.79; S, 9.51.

#### 3.2.3. 5-(4-Bromophenylamino)-3-phenyloxazolo[5,4-*c*]pyridazin-6(5*H*)-one (**4**)

A mixture of compound **1b** (0.01 mol), ethyl chloroformate (0.01 mol) and anhydrous potassium carbonate (0.04 mol) in dry acetone (30 mL) was refluxed for 20 h. The excess of solvent was evaporated then poured onto water. The solid obtained was filtered off and crystallized from ethanol to give compound **4**. Yield 61%, m.p. 173–175 °C. IR spectrum (ν, cm^−1^): 3228 (NH), 1715 (C=O). ^1^H-NMR (δ ppm): 4.41(s, 1H, NH; D_2_O exchangeable), 6.67(s, 1H, pyridazine-H), 7.26–8.26 (m, 9 H, Ar-H); MS, *m/z* (%): 382 (M^+^, 67), 384 (M^+^ + 2, 63). Analysis for C_17_H_11_BrN_4_O_2_ (383.21): calcd. C, 53.28; H, 2.89; Br, 20.85; N, 14.62. Found C, 55.97; H, 2.61; Br, 20.37; N, 14.35.

#### 3.2.4. 5-(4-Bromophenylamino)-3-phenyl-5*H*-imidazo[4,5-*c*]pyridazin-6(7*H*)-one (**5**)

A mixture of compound **4** (0.01 mol), ammonium acetate (0.03 mol) and a catalytic amount of ZnCl_2_ was fused in an oil-bath for 3 h. Then the mixture was poured onto water, and the resultant solid was filtered and recrystallized from dioxane to give **5**. Yield 51%, m.p. 180–182 °C. IR spectrum (ν, cm^−1^): 3220–3142 (2NH), 1688 (C=O). ^1^H-NMR (δ ppm): 4.32 (s, 1H, NH; D_2_O exchangeable), 6.40 (s, 1H, NH; D_2_O exchangeable), 6.82 (s, 1H, pyridazine-H), 7.66–8.35 (m, 9 H, Ar-H); MS, *m/z* (%): 381 (M^+^, 24), 383 (M^+^ + 2, 22). Analysis for C_17_H_12_BrN_5_O (382.22): calcd. C, 53.42; H, 3.16; Br, 20.91; N, 18.32. Found C, 53.15; H, 2.87; Br, 20.65; N, 18.01.

#### 3.2.5. 6-(4-Bromophenyl)-3-phenyl-6,7-dihydro-8*H*-pyridazino[3′,4′:4,5]imidazo[2,1-*b*][1,3,4]oxadiazin-8-one (**6**)

A mixture of compound **5** (0.01 mol), ethyl bromoacetate (0.01 mol) and anhydrous potassium carbonate (0.04 mol) in dry acetone (30 mL) was refluxed for 12 h; the excess of solvent was evaporated and the reaction mixture then poured onto water. The solid obtained was filtered off and crystallized from methanol to give **6**. Yield 44%, m.p. 199–201 °C. IR spectrum (ν, cm^−1^): 1703 (C=O). ^1^H-NMR (δ ppm): 4.09 (d, *J* = 11.63 Hz, 1H, CH_2_), 4.19 (d, *J* = 11.71 Hz, 1H, CH_2_), 6.57 (s, 1H, pyridazine-H), 7.33–8.42 (m, 9 H, Ar-H); MS, *m/z* (%): 421 (M^+^, 11), 423(M^+^ + 2, 9). Analysis for C_19_H_12_BrN_5_O_2_ (422.24): calcd. C, 54.05; H, 2.86; Br, 18.92; N, 16.59. Found C, 53.76; H, 2.55; Br, 18.60; N, 16.29.

#### 3.2.6. 4-(2-(4-Chlorophenyl)hydrazinyl)-3-hydrazinyl-6-phenylpyridazine (**7**)

A mixture of **2** (0.01 mol) and hydrazine hydrate (99%, 0.01 mol) in dioxane (20 mL) was heated under reflux for 6 h, the formed solid was filtered off and crystallized from ethanol to give **7**. Yield 55%; m.p. 102–104 °C; IR (ν, cm^−1^): 3350 (NH_2_), 3192–3135 (3NH); ^1^H-NMR (δ ppm): 4.38 (s, 1H, NH; D_2_O exchangeable), 4.45 (s, 1H, NH; D_2_O exchangeable), 5.14 (s, 2H, NH_2_; D_2_O exchangeable), 6.64 (s, 1H, pyridazine-H), 7.17–8.31 (m, 9 H, Ar-H), 8. 89 (s, 1H, NH; D_2_O exchangeable); MS, *m/z* (%): 326 (M^+^, 70), 328(M^+^ + 2, 24). Analysis for C_16_H_15_ClN_6_ (326.79): calcd. C, 58.81; H, 4.63; Cl, 10.85; N, 25.72. Found C, 58.56; H, 4.31; Cl, 10.57; N, 25.44.

#### 3.2.7. *N*-(4-Chlorophenyl)-3-methyl-6-phenylpyridazino[4,3-*e*][1,2,4]triazin-4(1*H*)-amine (**8**)

A solution of **7** (0.01 mol) and acetic anhydride (20 mL) was heated under reflux for 2 h. The solid obtained after cooling was crystallized from acetic acid to give **8**. Yield 46%; m.p. 125–127 °C; IR (ν, cm^−1^): 3180, 3132 (2NH); ^1^H-NMR (δ ppm): 2.38 (s, 3H, CH_3_), 4.35 (s, 1H, NH; D_2_O exchangeable), 6.7 (s, 1H, pyridazine-H), 7.27–8.35 (m, 9H, Ar-H), 8.96 (s, 1H, NH, D_2_O exchangeable); MS, *m/z* (%): 350 (M^+^, 14), 352 (M^+^ + 2, 4). Analysis for C_18_H_15_ClN_6_ (350.81): calcd. C, 61.63; H, 4.31; Cl, 10.11; N, 23.96. Found C, 61.37; H, 4.06; Cl, 9.86; N, 23.71.

#### 3.2.8. *N*,3-Bis(4-chlorophenyl)-6-phenylpyridazino[4,3-*e*][1,2,4]triazin-4(1*H*)-amine (**9**)

A mixture of **7** (0.01 mol) and *p*-chlorobenzaldehyde (0.01 mol) in ethanol (20 mL)/HCl (1 mL) was heated under reflux for 2 h, and then cooled. The separated solid was filtered, dried and crystallized from methanol to afford **9**. Yield 50%; m.p. 158–160 °C; IR (ν, cm^−1^): 3140; 3110 (2NH); ^1^H-NMR (δ ppm): 4.4(s, 1H, NH; D_2_O exchangeable), 6.66 (s, 1H, pyridazine-H), 7.10–8.81 (m, 13 H, Ar-H), 8.91 (s, 1H, NH; D_2_O exchangeable); MS, *m/z* (%): 446 (M^+^, 44), 448 (M^+^ + 2, 27), 450 (M^+^ + 4, 4). Analysis for C_23_H_16_Cl_2_N_6_ (447.32): calcd. C, 61.76; H, 3.61; Cl, 15.85; N, 18.79. Found C, 61.51; H, 3.35; Cl, 15.60; N, 18.63.

#### 3.2.9. 4-(4-Chlorophenylamino)-6-phenyl-1,2-dihydropyridazino[4,3-*e*][1,2,4]triazine-3(4*H*)-thione (**10**)

A mixture of **7** (0.01 mol), carbon disulfide (0.02 mol) and (0.01 mol) KOH in ethanol (50 mL) was refluxed for 9 h. The solvent was evaporated under vacuum, dissolved in hot water, and then the filtrate was neutralized with diluted hydrochloric acid. The precipitate was collected after washing with water several times. The product obtained was recrystallized from dioxane to give compound **10**. Yield 43%; m.p. 111–113 °C; IR (ν, cm^−1^): 3144–3160 (3NH), 1230 (C=S); ^1^H-NMR (δ ppm): 4.44 (s, 1H, NH; D_2_O exchangeable), 5.04 (s, 1H, NH; D_2_O exchangeable), 6.70 (s, 1H, pyridazine-H), 7.20–8.38 (m, 9H, Ar-H), 8. 86 (s, 1H, NH; D_2_O exchangeable); MS, *m/z* (%): 368 (M^+^, 56), 370 (M^+^ + 2, 20). Analysis for C_17_H_13_ClN_6_S (368.85): calcd. C, 55.36; H, 3.55; Cl, 9.61; N, 22.78; S, 8.69. Found C, 55.09; H, 3.39; Cl, 9.37; N, 22.51; S, 8.44.

#### 3.2.10. 5-(4-Chlorophenylamino)-7-(3,5-dimethoxybenzylidene)-3-phenyl-5*H*-pyridazino[3,4-*b*][1,4]thiazin-6(7*H*)-one (**11**)

A mixture of **3** (0.01 mol), chloroacetic acid (0.01 mol), anhydrous sodium acetate (0.01 mol) in glacial acetic acid/acetic anhydride (40 mL, 3:1) and 3,4-dimethoxybenzaldehyde (0.01 mol) was added. The reaction mixture was heated under reflux for 3 h, then cooled and poured onto water. The solid formed was collected by filtration and crystallized from dioxane to give compound **11**. Yield 50%; m.p. 106–108 °C; IR (ν, cm^−1^): 3150 (NH), 1697 (C=O); ^1^H-NMR (δ ppm): 3.85 (s, 3H, OCH_3_), 3.86 (s, 3H, OCH_3_), 4.45 (s, 1H, NH; D_2_O exchangeable), 6.82 (s, 1H, pyridazine-H), 7.38–7.90 (m, 12H, Ar-H), 8.33 (s, 1H, exocyclic vinylic-H); MS, *m/z* (%): 516 (M^+^, 47), 518 (M^+^ + 2, 16). Analysis for C_27_H_21_ClN_4_O_3_S (517.01): calcd. C, 62.73; H, 4.09; Cl, 6.86; N, 10.84; S, 6.20. Found C, 62.45; H, 3.79; Cl, 6.57; N, 10.61; S, 5.88.

#### 3.2.11. *N*-(4-Chlorophenyl)-3-(3,5-dimethoxyphenyl)-7-phenyl-2,3-dihydro-9*H*-isoxazolo[4,5-*b*]pyridazino[4,3-*e*][1,4]thiazin-9-amine (**12**)

A mixture of **11** (0.01 mol) and hydroxylamine hydrochloride (0.01 mol) was refluxed in pyridine (5 mL) for 10 h. The reaction mixture was cooled, poured onto water (100 mL) and neutralized with dilute HCl, the product was filtered, dried and crystallized from dioxane to give compound **12**. Yield 58%; m.p. 144–146 °C; IR (ν, cm^−1^): 3150, 3110 (2NH); ^1^H-NMR (δ ppm): 3.84 (s, 3H, OCH_3_), 3.85 (s, 3H, OCH_3_), 4.46 (s, 1H, NH; D_2_O exchangeable), 4.73 (s, 1H, isoxazole-H), 6.80 (s, 1H, pyridazine-H), 7.11–7.92 (m, 12H, Ar-H), 8.92 (s, 1H, NH; D_2_O exchangeable); MS, *m/z* (%): 531 (M^+^, 36 ), 533 (M^+^ + 2, 13). Analysis for C_27_H_22_ClN_5_O_3_S (532.03): calcd. C, 60.96; H, 4.17; Cl, 6.66; N, 13.16; S, 6.03. Found C, 60.47; H, 3.89; Cl, 6.41; N, 1.88; S, 5.77.

#### 3.2.12. *N*-(4-Chlorophenyl)-3-(3,5-dimethoxyphenyl)-7-phenyl-2,3-dihydropyrazolo[4,3-*b*]pyridazino[4,3-*e*][1,4]thiazin-9(1*H*)-amine (**13**)

A mixture of compound **11** (0.01 mol) and hydrazine hydrate (0.02 mol) was refluxed in ethanol (20 mL) for 5 h, then poured onto cold water. The solid substance was filtered off, dried and recrystallized from dioxane to give compound **13**. Yield 62%; m.p. 173–175 °C; IR (ν, cm^−1^): 3195–3111 (3NH); ^1^H-NMR (DMSO-*d*_6_, δ ppm): 3.80 (s, 3H, OCH_3_), 3.82 (s, 3H, OCH_3_), 4.43(s, 1H, NH, D_2_O exchangeable), 4.83 (s, 1H, pyrazole-H), 6.69 (s, 1H, pyridazine-H), 7.11–7.90 (m, 12H, Ar-H), 8.88 (s, 1H, NH, D_2_O exchangeable) 9.08 (s,1 H, NH, D_2_O exchangeable); MS, *m/z* (%): 530 (M^+^, 51), 532 (M^+^ + 2, 17). Analysis for C_27_H_23_ClN_6_O_2_S (531.04): calcd. C, 61.07; H, 4.37; Cl, 6.68; N, 15.83; S, 6.04. Found C, 60.77; H, 4.09; Cl, 6.41; N, 15.58; S, 5.76.

#### 3.2.13. 7-Amino-5-((4-chlorophenyl)amino)-9-(3,5-dimethoxyphenyl)-3-phenyl-5*H*-pyridazino[3,4-*b*]pyrido[2,3-*e*][1,4]thiazine-8-carbonitrile (**14**)

A mixture of **11** (0.01 mol), malononitrile (0.01 mol), and anhydrous ammonium acetate (0.02 mol) was refluxed in glacial acetic acid (40 mL) for 24 h. The reaction mixture was cooled and poured onto water. The formed solid was filtered off, dried and recrystallized from dioxane to give compound **14**. Yield 60%; m.p. 133–135 °C; IR (KBr, ν, cm^−1^): 3355 (NH2), 3170 (NH), 2222 (CN); ^1^H-NMR (δ ppm): 3.80 (s, 3H, OCH_3_), 3.81 (s, 3H, OCH_3_), 4.42 (s, 1H, NH; D_2_O exchangeable), 5.3 (s, 2H, NH_2_; D_2_O exchangeable), 6.79 (s, 1H, pyridazine-H), 7.36–7.89 (m, 12H, Ar-H); MS, *m/z* (%): 580 (M^+^, 27), 582 (M^+^ + 2, 9). Analysis for C_30_H_22_ClN_7_O_2_S (580.06): calcd. C, 62.12; H, 3.82; Cl, 6.11; N, 16.90; S, 5.53. Found C, 62.04; H, 3.77; Cl, 5.91; N, 16.78; S, 5.49.

#### 3.2.14. 5-((4-Chlorophenyl)amino)-9-(3,5-dimethoxyphenyl)-7-oxo-3-phenyl-6,7-dihydro-5*H*-pyridazino[3,4-*b*]pyrido[2,3-*e*][1,4]thiazine-8-carbonitrile (**15**)

A mixture of **11** (0.01 mol), ethyl cyanoacetate (0.01 mol) and ammonium acetate (0.01 mol) in *n*-butanol (40 mL) was refluxed for 10 h. The precipitate was filtered off, dried and recrystallized from dioxane to give compound **15**. Yield 66%; m.p. 256–258 °C; IR (ν, cm^−1^): 3130, 3166 (2 NH), 2221 (CN), 1680 (C=O); ^1^H-NMR (δ ppm): 3.83 (s, 3H, OCH_3_), 3.84 (s, 3H, OCH_3_), 4.40 (s, 1H, NH; D_2_O exchangeable), 6.74 (s, 1H, pyridazine-H), 7.31–7.90 (m, 12H, Ar-H), 8.59 (s, 1H, NH pyridine; D_2_O exchangeable); MS, *m/z* (%): 580 (M^+^, 46), 582 (M^+^ + 2, 16). Analysis for C_30_H_21_ClN_6_O_3_S (581.04): calcd. C, 62.01; H, 3.64; Cl, 6.10; N, 14.46; S, 5.52. Found C, 61.96; H, 3.51; Cl, 5.90; N, 14.29; S, 5.50.

### 3.3. Anti-Viral Bioassay

Cells: HEPG2 cells were propagated in DMEM medium. They were supplemented with 10% foetal bovine serum, 1% antibiotic-antimycotic mixture.

Viruses: a cell culture adapted strain of hepatitis A was provided by Dr. Ali Fahmy, Prof. of Virology at VACSERA (Cairo, Egypt). Virus was titrated to give final concentration 10^6^ PFU/mL.

Synthetic compounds preparation for bioassay: 10 mg of each compound was dissolved in 10% DMSO and 90% deionized water, decontaminated with 1% antibiotic-antimycotic mixture and stored in −20 °C.

#### 3.3.1. Cytotoxicity Assay

The cell culture safety doses of the dissolved synthetic compounds were determined by cell morphology technique [69]. A 96 well plate was seeded with HEPG2 cells and incubated overnight. Synthetic compounds were inoculated at concentrations of 5, 10, 15, 20, 25 and 30 µg/mL and observed microscopically for any morphological changes after 24 h incubation at 37 °C in a humidified incubator with 5% CO_2_.

#### 3.3.2. Plaque Infectivity Count Assay

Plaque infectivity count assay is the most widely accepted method for determining the % inhibition of virus as a result of being subjected to a given material [70]. A 12 well plate was cultivated with the HEPG2 cells (10^5^ cell/mL) and incubated for overnight at 37 °C. Virus was and mixed with the safe concentrations of each compound and incubated for 1 h at 37 °C. Growth medium was removed from the multi-well plate and virus-compound mixture was inoculated in plate wells. After 1 h contact time for virus adsorption, 1 mL of 2× DMEM medium 2% agarose overlaid the cell sheet. The plates were left to solidify and incubated at 37 °C until the development of the viral plaques. Formalin was added for two hours then plates were stained with crystal violet staining solution. Control virus and cells were treated identically without compounds. Viral plaques were counted and the percentage of virus reduction was calculated.

#### 3.3.3. Mechanism of Virus Inhabitation

The possible mechanism of HAV inhibition by the compounds was studied at three different levels:

##### Extract Affects Viral Particle Itself (Virucidal)

The virucidal assay [71] was carried out in a 12 well plate where HEPG2 cells were cultivated (10^5^ cell/mL) overnight at 37 °C. A volume of 100 µL serum free DMEM containing 10^6^ PFU HAV was added to the concentration of compound resulting in viral inhibition. After 1 h incubation, the mixture was diluted using serum free medium 3 times, each is 10 fold, that still allows existence of viral particles to grow on HEPG2 cells, but leaves nearly no extract (100 µL of each dilution were added to the HEPG2 cell monolayer). After 1 h contact time, a DMEM overlay with 2% agarose was added to the cell monolayer. Plates were left to solidify then incubated at 37 °C to allow formation of viral plaques and completed as previously mentioned.

##### Extract Binds to Cell Receptor Preventing Viral Adsorption (Early Replication Step)

Viral adsorption [72] HepG2 cells were cultivated in a 12 well plate (10^5^ cell/mL) and incubated overnight at 37 °C. Compound was applied at different concentrations in 200 µL medium without serum and co-incubated with the cells for 2 h at 4 °C. Unadsorbed compound was removed by washing cells three successive times with serum free-medium. HAV virus (diluted to 10^4^ PFU/well) was then co-incubated with the pretreated cells for 1 h followed by adding 3 mL DMEM with 2% agarose. Plates were left to solidify then incubated at 37 °C to allow formation of viral plaques and completed as previously mentioned.

##### Extract Affects One of the Enzymes inside the Cell Needed by the Virus to Complete Its Replication Cycle (Late Replication Step)

A viral replication assay [73] was carried out in a 12 well plate where HEPG2 cells were cultivated (10^5^ cell/mL) and incubated overnight at 37 °C. Virus was diluted to give 10^4^ PFU/well, applied directly to the cells and incubated for 1 h at 37 °C. Unadsorbed viral particles were removed by washing cells three successive times by serum free-medium. Compound was applied at different concentrations, after 1 h contact time, 2 mL of DMEM medium supplemented with 2% agarose was added to the cell monolayer. Plates were left to solidify and incubated at 37 °C till appearance of viral plaques and completed as previously mentioned.

## 4. Conclusions

Pyridazines have attracted attention because of their easy functionalization at various ring positions, which makes them attractive synthetic building blocks for designing and developing novel pyridazine-containing agents. The incorporation of substituents in the pyridazine ring or as a fused component often leads to incredibly diverse biological activity. The biological profile of these new generations of pyridazines is of great interest. This study highlights the status of pyridazinones in the development of novel pyridazinone-based drug candidates as anti-HAV agents. At least one compound (compound **10**) has high anti-HAV activity as a result of having a direct effect on the virus (virucidal effect) and also has mild effect on viral replication, so it could be used as a precursor for anti-HAV drugs. The importance of this compound comes from the current lack of antiviral drugs for HAV and high fatality rates resulting from HAV coinfection with chronic HBV or HCV patients.

## Figures and Tables

**Table 1:** Safe doses of each compound.

Compounds	Concentrations (µg/mL) *					
	5	10	15	20	25	30
**2**	-	-	-	+2	+3	+3
**3**	-	-	-	-	-	-
**4**	-	-	-	-	-	-
**5**	-	-	-	-	-	-
**6**	-	-	-	-	-	-
**8**	-	-	-	+1	+2	+4
**9**	-	-	-	+1	+2	+4
**10**	-	-	-	+2	+3	+4
**11**	-	-	-	-	-	+1
**12**	-	-	-	-	-	-
**13**	-	-	-	+1	+2	+3
**14**	-	-	-	-	-	-
**15**	-	-	+1	+3	+4	+4

* Safe doses, +1: 25% of cell sheet was affected, +2: 50% of cell sheet was affected, +3: 75% of cell sheet was affected, +4: 100% of cell sheet was affected.

**Table 2:** The antiviral activity of the synthesized compounds against HAV.

Compound	Concentration (µg/mL)	% Inhibition
**2**	10	13
	15	29
**3**	20	19
	25	66
**4**	20	56
	25	73
**5**	20	30
	25	62
**6**	20	0
	25	0
**8**	10	26
	15	65
**9**	10	37
	15	50
**10**	10	100
	15	100
**11**	20	50
	25	75
**12**	20	42
	25	37
**13**	10	33
	15	4
**14**	20	46
	25	76
**15**	10	0
	15	38
